# Ethnic Disparities in Utilization of Maternal and Child Health Services in Rural Southwest China

**DOI:** 10.3390/ijerph17228610

**Published:** 2020-11-19

**Authors:** Chaofang Yan, Charuwan Tadadej, Kanittha Chamroonsawasdi, Natkamol Chansatitporn, John FC Sung

**Affiliations:** 1 Department of Public Health Administration, Faculty of Public Health, Mahidol University, Bangkok 10400, Thailand; chaofangyan@hotmail.com; 2 Department of Family Health, Faculty of Public Health, Mahidol University, Bangkok 10400, Thailand; kanittha.cha@mahidol.ac.th; 3 Department of Biostatistics, Faculty of Public Health, Mahidol University, Bangkok 10400, Thailand; nutkamol.cha@mahidol.ac.th; 4 Institute of Health and Development Studies, School of Public Health, Kunming Medical University, Kunming 650500, China; fcsung1008@yahoo.com

**Keywords:** ethnic disparity, utilization, maternal and child health services, China

## Abstract

Background: Studies in China on ethnic disparities in access to health care in remote and rural population remain insufficient. This study aimed to assess the disparities in utilization of maternal and child health (MCH) services, including antenatal care (ANC), hospital birth, child growth monitoring, and immunization compliance between Han and ethnic minority women in Yunnan Province. Methods: A multi-stage sampling scheme was used to randomly recruit women from 40 townships in 14 remote prefectures of extremely remote areas in Yunnan. From birth records, we identified and recruited 303 Han women and 222 ethnic minority women who had given birth to a child within 3 years for an interview. Results: Overall, 96% of women used the ANC checkups and more than 95% had infants born in hospitals. However, the proportion of women compliant with early ANC visits (having antenatal care in the first trimester) was 22.5% lower in minority women than in Han women (61.3% vs. 83.8%, *p* < 0.001) with an adjusted odds ratio (aOR) of 2.04 (95% confidence interval (CI) of 1.13–3.66) for the minority group. The proportion of children under one year old with immunizations completed in a timely manner was also lower in minority families than in Han families (80.2% vs. 86.8%, *p* < 0.05) with an aOR of 1.99 (95% CI = 1.16–3.40). Conclusions: Ethnic disparities remain in utilization of early ANC visits and timely immunization completion for newborns. Ethnic minority women tended to lag behind for both. Further intervention should focus on assisting minority women living in extremely rural areas to comply with the MCH policy. Culturally-sensitive policies and skills are needed, and priority should be given to improve utilization of early ANC and timely immunization completion.

## 1. Introduction

Women and children are at a crucial position in social development. The United Nations (UN) has highlighted strategies to promote the health of women and children in the Millennium Development Goals (MDG) 4 and 5 from 2000 to 2015, and in Sustainable Development Goal (SDG) 3 from 2016 to 2030. Substantial progress in maternal and child survivals has been made since the 1990s [1]. Timely maternal and child health (MCH) services utilization is vital to reduce both the maternal and child deaths [2,3,4,5]. The mediation of the MDG has increased utilization of antenatal care (ANC) services to 44% and assisted delivery to 12%. The UN efforts have thus reduced maternal deaths by 44% from 283.2 per 100,000 live births in 1990 [4,6]. The coverage for priority interventions among disadvantaged populations in a country is an indication of the strength of the health system [7].

Reducing MCH disparity has been a crucial issue across the whole nation in China, a developing country with the largest population in the world [8,9,10,11]. The Chinese government has taken a series of actions in order to increase the coverage of the interventions of MCH among disadvantaged populations in less developed regions [12,13,14]. For example, from 1996 to 1999 the government started a World Bank loan program, the Poverty Alleviation Fund for Maternal and Child Health. The program aimed to reduce maternal mortality rate (MMR) and infant mortality rate (IMR) by assisting 5% of poor families with their payment of the MCH services in five western provinces [12,15]. From 2000 to 2008, the program of Reducing Maternal Mortality and Eliminating Neonatal Tetanus was launched to reduce MMR, mainly targeting 378 counties in rural areas [14]. The Basic Public Health Service (BPHS) project, launched in 2009, is one of the most effective policies for providing and promoting residents with equal access to basic public health services. This government-funded project includes nine service categories, including one reform to improve MCH services [13,16]. After years of effort, the urban–rural disparity of MMR in China has been greatly narrowed. The urban-to-rural ratio of MMR reduced from 1:2.2 in 1990 to 1:1.3 in 2018 (15.5 vs. 19.9 deaths per 100,000 livebirths) [10]. The western part of China is less developed than the eastern part of China; the eastern-to-western region ratio of MMR reduced from 1:4.7 in 1996 to 1:2.3 (10.9 vs. 25.2 per 100,000 livebirths) in 2018. The project also reduced the urban–rural ratio of the under-5 mortality from 1:3.4 in 1991 to 1:2.3 in 2018 (4.4 vs. 10.2 per 1000 livebirths). The gap in under-5 mortality rates between east and west regions was reduced to 8.5‰ in 2018 from 66.5‰ in 1991 [10].

The MCH disparities between east and west China, or between urban and rural, have been explored and well documented. However, studies evaluating the ethnic disparities with robust methods remain severely insufficient in China [17]. The Lancet–Lowitja Institute Global Collaboration study has shown that Yunnan province and Tibet Autonomous Region in China have not yet reached the UN SDG for underserved populations [18]. Some studies related to ethnic populations have reported health coverage and health outcomes based on the whole population from the Autonomous Region rather than classified by ethnic type [8,19]. No study has compared differences of MCH between Han and ethnic minorities at a provincial level, because the medical records and vital statistics were rarely classified by ethnicity in the official health statistics system. Most studies have emphasized the improvement of health data collection for ethnic minorities in China [17,18,20].

China is populated with multiple ethnic groups with high cultural and language diversities; these consist of the Han majority accounting for 91.5% of the total population and 55 minority groups accounting for the rest of the population (8.5%). The 2010 census estimated that 114 million people in China were ethnic minorities. Three-quarters (71.4%) of all ethnic minorities live in the remote southern and western regions of China, including Yunnan Province [21].

**Figure 1:**
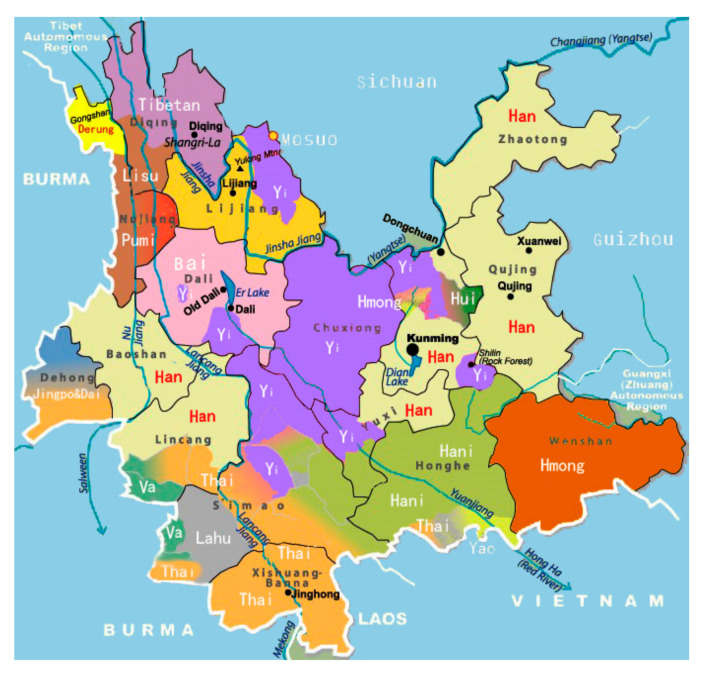
Ethnic diversity of Yunnan Province, China.

Yunnan Province is located in Southwestern China, bordering Myanmar in the west, and Laos and Vietnam in the south. It is one of the poorest remote provinces and has the highest ethnic diversity in China (Figure 1), including 8 ethnic autonomous prefectures and 29 ethnic autonomous counties. Among 56 recognized ethnic groups in China, Yunnan Province has recognized at least 25 ethnic minority groups, making up 33.6% of the 48 million population in the province in 2018. Yunnan is situated in mountainous land, with only 6% of plain areas suitable for cultivation [22]. The provincial authority has endeavored to carry out the ambitious provincial goal since 2010 for reducing MMR to 25/100,000 and IMR to 11 per 1000 live births by 2020, then reducing the MMR to 12/100,000 and the IMR to 5 per 1000 live births by 2030, based on the MMR 37.3/100,000 and the IMR 15.2 per 1000 live births in 2010 [23,24]. For better utilization of MCH services, pregnant women are officially encouraged to complete at least five ANC with the first ANC in the first trimester, hospital delivery, having their children under one year old checked for growth monitoring at least four times, and getting their children vaccinated in a timely manner according to the National Immunization Program [25].

As a province with typical ethnic minorities, no study has yet compared the compliance of MCH care services between Hans and ethnic minorities in Yunnan. Therefore, we conducted a survey in remote rural areas in the province to assess the disparities in utilization of MCH services between Han and the ethnic minority mothers.

## 2. Materials and Methods

### 2.1. Study Design and Participants Recruitment

This study was a cross-sectional survey with a comparison between Han and ethnic minorities. Women who had given birth within the past three years at the time of the survey and had been residing in the selected township in Yunnan for at least three years were included in the study. This inclusion criteria ensured that these women could provide the health service information for both mothers and children, such as the information of growth monitoring and immunization. Women who worked and used health services out of the selected township were excluded to show the effect of ethnicity in the same context. A conceptual framework, adapted from the model of access to medical care by Andersen [26], was used to examine the access to MCH services and to analyze the factors affecting access to MCH services.

The sample size was calculated based on detecting a significant proportional difference of hospital births between ethnic minorities and the Han group at a 5% level with 80% power of a two-sided test. Previous studies in Yunnan Province have reported that the hospital birth rates were 81% (512/631) in minority women and 91% (476,132/523,223) in Han women [27,28]. These data were used to calculate the minimum sample size being at least 188 subjects in each group for this study.

A multi-stage sampling method was used to select the study sample. Firstly, we excluded the provincial capital Kunming to eliminate the effects of economic inequality and Nujiang prefectures that are difficult to reach by automobile transportation. For the rest of the 14 prefectures, 1 county was randomly selected from each prefecture. In each selected county, after excluding the township in which the county government was located, we randomly selected a township. Based on the birth records from the township hospital in the selected township, 40 women with a birth within the past three years at the time of the survey were randomly selected from each township, resulting in a total of 560 women selected as potential participants. For participants with more than one pregnancy and delivery, information from the last pregnancy was collected and used.

This study was approved by the ethics committee of the Faculty of Public Health, Mahidol University (COA No. MUPH 2014-214; 24 November 2014). All participants were clearly informed of their rights and any risks associated with participation. Verbal consent was obtained from all interview participants.

### 2.2. Study Procedure and Data Collection

We applied the Andersen health behavior model to establish a structured questionnaire for data collection of the survey to investigate MCH services associated with predisposing (demographic and social) factors, enabling (economic) factors, and need (health outcomes) factors [26]. The questionnaire was reviewed by experts in MCH, social medicine, and health management.

The questionnaire asked for four types of information. The first type regarded predisposing factors including the general characteristics of respondents, comprising age of the woman, age of the child, sex of the child, parity, ethnicity, language ability, education, and knowledge on MCH. The second part regarded enabling factors: the family income, family size and health insurance, the usual health facility, and travel time to the nearest health facility for childbirth. The third part regarded need factors: the woman’s health status during the perinatal period, previous obstetric problems, child health status, preterm delivery, and birth weight of the child. The fourth part asked for information about MCH utilization, including ANC utilization, timing for ANC visits, hospital birth, delivery type, child growth monitoring services, timing for child growth monitoring visits, and immunization services.

Before the actual data collection, the questionnaire was pre-tested on 20 women from villages of the study area. The reliability of scaling variables was assessed by calculating Cronbach’s coefficient alpha (α = 0.716), which indicated that the measures had fairly high levels of reliability. The questionnaire was administered in person by trained interviewers at each participant’s household. We trained 32 medical students who could speak the local dialog as interviewers. A two-day interview workshop was given to them before conducting the survey.

After excluding 22 women who refused interviews and 13 women who could not complete the questionnaires, information was obtained from 525 households in total. The ethnic minorities included 15 ethnicity groups of Yi, Hani, Thai, Zhuang, Hmong, Bai, Yao, Hui, Lisu, Va, Lahu, Nu, Jingpo, Achang, and Paijiao.

### 2.3. Statistical Analysis

Eligible questionnaires were edited and coded, and the data were entered and processed using SPSS version 19. Data analysis first compared distributions of participants’ characteristics in predisposing factors, enabling factors, and need factors between Han and ethnic minority participants. Utilization of MCH care (ANC visit, infant delivery, growth monitoring, and immunization) was presented by numbers and proportions. Chi-square test was used to examine the difference between the two groups. Logistic regression analysis was further used to calculate odds ratio (OR) and 95% confidence interval (CI) of inadequate utilization of MCH care for the ethnic minority women, compared to Han women. The adjusted odds ratio (aOR) was estimated with a multivariable by three models: after controlling for predisposing factors, after controlling for predisposing and enabling factors, and after controlling for predisposing, enabling, and need factors.

## 3. Results

### 3.1. Socio-Demographic Profile

With response rates of 97.1% and 89.5% in Han and ethnic minorities, respectively, 303 Han women and 222 minority women completed the questionnaire interviews. The average ages of participants in both Han and ethnic minority groups were approximately similar at 26 years old. The Han group had higher education and better Mandarin language ability, with higher income, than the ethnic minority group. Ethnic minority women were more likely (69.4%) to be married to ethnic minority husbands (refer to Husband’s Ethnicity type) than were Han women (8.3%), and they had a larger family size than Han women did. Ethnic minority women needed more travel time to reach their nearest health care facility and perceived that their children had a poor health status. (Table 1).

### 3.2. Comparison of MCH Utilization

More than 90% of women had at least one ANC visit and nearly 60% made five visits during their pregnancy (Table 2). The Han women had a higher adherence to the recommended 5-visit schedules than did ethnic minority women by making their first ANC visit in the first trimester (58.1% vs. 43.2%, *p* < 0.001). However, making an early ANC visit was 22.5% lower in minority women than in Han women (61.3 vs. 83.8%). Over 95% of participants had their infants delivered at health institutions, with a higher incidence of Caesarean delivery in Han women (26.1% vs. 20.3%, p 0.12). However, a lower proportion of ethnic minority women completed the required vaccinations for children within the first year of age than Han women (80.2% vs. 86.8%, *p* < 0.05).

### 3.3. Odds Ratio of MCH Services Utilization

Table 3 shows that, compared with the Han women, the minority women were at higher risks for inadequate antenatal care, with significant unadjusted ORs of 3.59 (95% CI = 1.37–9.40) for failing to make at least one ANC visit during the pregnancy, of 3.28 (95% CI = 2.00–4.75) for failing to make an early ANC visit in the first trimester, and of 1.82 (95% CI = 1.28–2.58) for failing to make at least 5 ANC visits with an early ANC visit in the first trimester. However, only the risk of failing to make an early ANC visit remained significant after controlling for predisposing, enabling, and need factors, with an aOR of 2.04 (95% CI = 1.13–3.66). The ethnic minorities were also at a higher risk of not receiving vaccinations for children with all doses in a timely manner, with an aOR of 1.99 (95% CI = 1.16–3.40) after controlling for all covariables.

## 4. Discussion

This study aimed to assess the disparities in the utilization of MCH services between Han and ethnic minority mothers in remote rural areas. Theoretically, minority women and Han women could receive similar care even in the remote rural areas. We found that gaps of using MCH services still existed in ANC utilizations, significant for the early ANC visit in the mountainous rural region of Yunnan. The pregnant women from minority families were less likely than Han women to make the first ANC within the first 12 gestational weeks. Previous studies have rarely measured the disparities between the Han and ethnic minority populations in pregnancies, although some studies have reported the rates of the early ANC visits related to ethnic minority population in other provinces [29,30,31,32,33]. The timing of the first antenatal care visit is an important compliance measure to ensure optimal pregnancy outcomes for women and children [4,34]. The early antenatal care visit also has been used as an indicator to measure the quality of MCH services since launching the health care reform in 2009 in China [25]. This finding might imply the need of exploring the cultural factors for early ANC visits among ethnic minority women. Our study also showed the need to develop additional culture-sensitive policy to eliminate inequality and deliver cost effective MCH interventions.

Further, compared to Han mothers, the ethnic minority mothers were also less likely to have their children immunized in a timely manner, which is crucial for preventing childhood diseases. Studies have consistently reported a poor compliance with immunization services for children of ethnic minorities in China [17,35,36,37]. With an investment of USD $120 billion, the universal health insurance coverage increased to 95% of the population in 2019 from 30% in 2003 [38]. The economic barriers to accessing ANC, hospital births, and immunization services for rural populations have been removed, yet disparities were still observed.

This study found that hospital birth and caesarean delivery between Han and ethnic minority women were not significantly different. This was likely because of the compliance to the UN’s MDG and SDG strategies to promote the health of women and children. In China, like many developing countries, the economic level plays an important role in health service accessibility, especially in remote rural areas where many ethnic minorities live. Over the past 20 years, the Chinese Government has introduced several strategies aimed specifically at reaching underserved populations in rural areas. In 2003, a voluntary health insurance program, New Cooperative Medical Scheme (NCMS), was launched for rural residents. This heavily subsidized program has increased outpatient and inpatient services utilization by reducing the health service cost with a sliding fee discount of 30% to 80%, varying by region annually [39,40]. In addition to the major support from the central authority, some of the local health administrations also received support from international organizations such as the United Nations International Children’s Emergency Fund (UNICEF) [12,15,41]. The administrations were thus capable of conducting pilot studies using poverty alleviation funds to help low income pregnant women adhere to MCH services in a timely manner in some identified poverty-stricken counties.

Similarly, there were also no significant differences in using service of growth monitoring for children under 1 year old between Han and ethnic minority families in this study. The BPHS project of new medical reform policies has made a great contribution to promoting equal access to basic public health services since it was launched in 2009 [42]. This government-funded project included nine service categories, one of which was to improve infant cares. The project encouraged all families having their children under 1 year old to complete “four times-routine examination at the 3, 6, 8, and 12 months of age” to monitor growth and development free of charge. Further, all MCH physicians are required to have standardized training and to deliver the same maternal services for rural and urban populations [25].

As a whole, we demonstrated a substantial improvement in utilization of MCH services among all women in this study compared with the utilizations in the 1990s, providing valid information on the improvement of MCH services in remote rural areas. Overall, 96% of pregnant women used the antenatal checkups at least once in the present study, compared to 58.07% in 1992 in the whole Yunnan province. The compliance with at least five antenatal checkups improved to 60.6% from 23.78% in 1992. The greatest improvement is that the hospital birth increased to 95.8% from only 30.33% in 1990. More importantly, vital statistics data showed the MMR had a great decline from 115.29 per 100,000 livebirths in 1992 to 17.72 per 100,000 livebirths in 2018, and the infant mortality rates declined from 46.38‰ in 1992 to 5.85‰ in 2018 in Yunnan Province [43,44]. All the policies such as the NCMs and BPHS contribute to this great progress. The Targeted Poverty Alleviation Project was recently launched to further increase accessibility to local health care without charge for families officially registered as Poverty-Stricken Households [45]. We anticipate this effort will further reduce the ethnic disparities in MCH services utilization in rural China. However, most current policies tend to invest more money to reduce health inequality but have less emphasis on cultural barriers, transportation barriers, and development of the health workforce in extremely rural areas [46,47]. Minority women living in these areas deserve further intervention if the highest ethnic diversity province wants to reach the goals of MMR of 12/100,000 and the IMR of 5 per 100 live births by 2030 [24].

This study has a few limitations. This study excluded women who had experienced an abortion or had children who died under the age of 3 years. Their utilization of MCH services is not clear. Among the 560 women randomly invited to participate in this study, 35 women were not interviewed. These women were less likely to be compliant with required MCH checkups, and their information was not included in this study. Furthermore, information on MCH services was collected from women within 3 years of the childbirth at the time of survey, the self-reported data throughout the period might vary slightly among women. However, interviewers conducted the survey following strict protocols. The response bias was likely unintentional and would not adversely affect the survey outcome.

## 5. Conclusions

This study found that gaps of using MCH services between ethnic minorities and Han women exist in ANC utilization and timely immunization completion in China. Ethnic minority women tended to lag behind in utilization of early ANC, and in timely immunization completion for their children under one year old. However, there were no significant differences in hospital birth, caesarean delivery, and growth monitoring for children under 1 year old between Han and ethnic minority women in this study. China’s political commitment to poverty reduction for compliance with the UN MDG and SDG might have contributed to the improvement. A heavily subsidized NCMS and the BPHS of new medical reforms might also play an important role in promoting equal access to MCH services. Minority women living in extremely rural areas deserve further intervention. Culturally-sensitive policies and skills are needed to target ethnic populations to improve their access to MCH services in order to continue decreasing the urban–rural and ethnic disparities. Priority should be given to improve utilization of early ANC and timely immunization completion.

## Figures and Tables

**Table 1:** Sociodemographic characteristics of study subjects in Yunnan.

Characteristics	Total N = 525	Han N = 303	Minorities N = 222	*p*-Value
	n (%)	n (%)	n (%)	
**Predisposing factors**				
Age, years	26 ± 4.6	26.1 ± 4.7	25.9 ± 4.6	0.48
Husband’s Ethnicity				<0.001
Han	346 (65.9)	278 (91.7)	68 (30.6)	
Ethnic minority	179 (34.1)	25 (8.3)	154 (69.4)	
Family size	5.1 ± 1.4	4.9 ± 1.4	5.2 ± 1.4	0.04
Parity				0.49
1	297 (56.6)	174 (57.4)	123 (55.4)	
2	194 (37.0)	109 (36.0)	85 (38.3)	
≥3	34 (6.5)	20 (6.6)	14 (6.4)	
**Enabling factors**				
Women’s Education				0.001
≤6 years	160 (30.5)	78 (25.8)	82 (36.9)	
7–9	241 (45.9)	138 (45.5)	103 (46.4)	
≥10	124 (23.6)	87 (28.7)	37 (16.7)	
Husband’s Education				<0.001
≤6 years	112 (21.3)	51 (16.8)	61 (27.5)	
7–9	283 (53.9)	155 (51.2)	128 (57.7)	
≥10	130 (24.8)	97 (32.0)	33 (14.9)	
Fluent in Mandarin				<0.001
None/Partly	29 (5.5)	2 (0.7)	27 (12.2)	
Yes	496 (94.5)	301 (99.3)	195 (87.8)	
Average annual per capita income, CNY ^1^				0.008
<2800	235 (44.8)	127 (41.9)	119 (53.6)	
≥2800	181 (34.5)	176 (58.1)	103 (46.4)	
Median	2800	3000	2500	
Health Insurance Status				0.88
Public (NCMS ^2^)	515 (98.1)	167 (98.0)	218 (98.2)	
Private/None	10 (1.9)	136 (2.0)	4 (1.8)	
Travel to nearest health facility				0.01
<30 min	176 (33.5)	94 (31.0)	82 (36.9)	
30–59 min	199 (37.9)	131 (43.2)	68 (30.6)	
≥60 min	150 (28.6)	78 (25.7)	72 (32.4)	
**Need factors**				
Perceived health status of mother				0.57
Poor/very poor	18 (3.4)	11 (3.7)	7 (3.2)	
Fair	125 (23.8)	72 (23.8)	53 (23.9)	
Good/Excellent	382 (72.8)	220 (72.6)	162 (73.0)	
Have any previous obstetric problem				0.60
Yes	48 (9.1)	26 (8.6)	22 (9.9)	
No	477 (90.9)	277 (91.4)	200 (90.1)	
Perceived health status of child				0.04
Poor/Very Poor	34 (6.5)	17 (5.7)	17 (7.7)	
Fair	98 (18.7)	54 (17.8)	44 (19.8)	
Good/Excellent	393 (74.8)	232 (76.6)	161 (72.5)	
Birth weight of Child	3210 ± 526	3230 ± 32	3183 ± 33	0.31

^1^ CNY, Chinese yuan, 1 USD = 6.2 CNY; ^2^ NCMS, New rural cooperating medical scheme.

**Table 2:** Comparison on utilization of maternal and child health care between Han and ethnic minority women.

Type of MCH ^1^ Services	Total	Han	Minorities	X2	*p*
	N = 525	N = 303	N = 222		
	n (%)	n (%)	n (%)		
Antenatal care					
ANC ^2^ at least 1 visit	504 (96.0)	297 (98.0)	207 (93.2)	7.61	<0.05
ANC at least 5 visits	318 (60.6)	192 (63.4)	126 (56.8)	2.34	0.13
Early ANC visit ^3^	390 (74.3)	254 (83.8)	136 (61.3)	27.38	<0.001
ANC at least 5 visits with an early ANC visit	272 (51.8)	176 (58.1)	96 (43.2)	11.3	<0.05
Hospital births					
Hospital birth	503 (95.8)	292 (96.4)	211 (95.0)	0.56	0.45
Caesarean Delivery	124 (23.6)	79 (26.1)	45 (20.3)	2.39	0.12
Growth monitoring services for children within the first year of age	231 (44.0)	126 (41.6)	105 (47.3)	1.70	0.19
Use at least 4 times	231 (44.0)	126 (41.6)	105 (47.3)	1.70	0.19
Immunization					
Vaccinated all doses	520 (99.0)	300 (99.0)	220 (99.1)	0.01	0.91
Vaccinated all doses timely	441 (84.0)	263 (86.8)	178 (80.2)	4.18	<0.05

^1^ MCH, maternal and child care; ^2^ ANC, antenatal care; ^3^ Early ANC visit, having antenatal care in the first trimester.

**Table 3:** Ethnic minority women compared to Han women odds ratio (OR) of inadequate MCH utilization and adjusted odds ratio (aOR) controlling for predisposing, enabling, and need factors.

Type of MCH Services	Base Model, OR (95% CI)	Predisposing Factors Model, OR (95% CI)	Predisposing-Enabling Factors Model, OR (95% CI)	Predisposing-Enabling-Need Factors Model, OR (95% CI)
Antenatal care				
ANC at least 1 visit	3.59 (1.37–9.40) *	1.80 (0.51–6.31)	1.62 (0.36–7.38)	1.88 (0.42–8.51)
ANC at least 5 visits	1.32 (0.93–1.88)	0.96 (0.60–1.53)	0.97 (0.59–1.60)	1.01 (0.61–1.67)
Early ANC visit	3.28 (2.00–4.75) *	1.81 (1.05–3.15) *	1.91 (1.07–3.41) *	2.04 (1.13–3.66) *
ANC at least 5 visits with an early ANC visit	1.82 (1.28–2.58) *	1.27 (0.81–2.00)	1.34 (0.83–2.17)	1.42 (0.87–2.32)
Hospital birth				
Hospital birth	1.38 (0.59–3.25)	1.00 (0.32–3.14)	0.90 (0.23–3.90)	0.95 (0.25–3.66)
Caesarean Delivery	0.72 (0.48–1.09)	0.71 (0.42–1.23)	0.76 (0.44–1.31)	0.77 (0.44–1.33)
Growth monitoring services for children within the first year of age				
Use at least 4 times	0.79 (0.56–1.12)	0.70 (0.44–1.10)	0.65 (0.41–1.05)	0.65 (0.41–1.05)
Immunization services				
Vaccinated all doses	0.91 (0.15–5.49)	1.68 (0.21–13.6)	2.47 (0.19–31.4)	2.47 (0.20–31.0)
Vaccinated all doses timely	1.63 (1.02–2.60) *	1.73 (1.03–2.89) *	1.79 (1.06–3.04) *	1.99 (1.16–3.40) *

Note: The Predisposing Factors Model controlled for husband ethnicity and family size. Predisposing-Enabling Factors Model controlled for husband ethnicity, family size, education, husband education, Mandarin language ability, income, and travel time. The Predisposing-Enabling-Need Factors Model controlled for husband ethnicity, family size, education, husband education, Mandarin language ability, income, travel time, and perceived child health, which were significant predictors. * *p* < 0.05.

## Abbreviations

aOR	adjusted odds ratio
ANC	antenatal care
BPHS	basic public health service
CI	confidence interval
IMR	infant mortality rate
MCH	maternal and child health
MDG	Millennium Development Goal
MMR	maternal mortality rate
NCMS	New Cooperative Medical Scheme
OR	odds ratio
SDG	Sustainable Development Goal
UNICEF	United Nations International Children’s Emergency Fund
